# Can a Dinosaur Think? Implementation of Artificial Intelligence in Extracorporeal Shock Wave Lithotripsy

**DOI:** 10.1016/j.euros.2021.02.007

**Published:** 2021-03-21

**Authors:** Sebastien Muller, Håkon Abildsnes, Andreas Østvik, Oda Kragset, Inger Gangås, Harriet Birke, Thomas Langø, Carl-Jørgen Arum

**Affiliations:** a Department of Health Research, SINTEF Digital, Trondheim, Norway; b Department of Circulation and Medical Imaging, Norwegian University of Science and Technology, Trondheim, Norway; c Medical School, Norwegian University of Science and Technology, Trondheim, Norway; d Department of Radiology, St. Olavs Hospital, Trondheim University Hospital, Trondheim, Norway; e Department of Surgery, St. Olavs Hospital, Trondheim University Hospital, Trondheim, Norway; f Department of Clinical and Molecular Medicine, Norwegian University of Science and Technology, Trondheim, Norway; g Department of Urology, Skane University Hospital, Malmö, Sweden; h Department of Translational Medicine, Lund University, Malmö, Sweden

**Keywords:** Extracorporeal shockwave lithotripsy, Kidney stones, Artificial intelligence, Machine learning, Neural network

## Abstract

**Background:**

Extracorporeal shock wave lithotripsy (ESWL) of kidney stones is losing ground to more expensive and invasive endoscopic treatments.

**Objective:**

This proof-of-concept project was initiated to develop artificial intelligence (AI)-augmented ESWL and to investigate the potential for machine learning to improve the efficacy of ESWL.

**Design, setting, and participants:**

Two-dimensional ultrasound videos were captured during ESWL treatments from an inline ultrasound device with a video grabber. An observer annotated 23 212 images from 11 patients as either in or out of focus. The median hit rate was calculated on a patient level via bootstrapping. A convolutional neural network with U-Net architecture was trained on 57 ultrasound images with delineated kidney stones from the same patients annotated by a second observer. We tested U-Net on the ultrasound images annotated by the first observer. Cross-validation with a training set of nine patients, a validation set of one patient, and a test set of one patient was performed.

**Outcome measurements and statistical analysis:**

Classical metrics describing classifier performance were calculated, together with an estimation of how the algorithm would affect shock wave hit rate.

**Results and limitations:**

The median hit rate for standard ESWL was 55.2% (95% confidence interval [CI] 43.2–67.3%). The performance metrics for U-Net were accuracy 63.9%, sensitivity 56.0%, specificity 74.7%, positive predictive value 75.3%, negative predictive value 55.2%, Youden’s *J* statistic 30.7%, no-information rate 58.0%, and Cohen’s κ 0.2931. The algorithm reduced total mishits by 67.1%. The main limitation is that this is a proof-of-concept study involving only 11 patients.

**Conclusions:**

Our calculated ESWL hit rate of 55.2% (95% CI 43.2–67.3%) supports findings from earlier research. We have demonstrated that a machine learning algorithm trained on just 11 patients increases the hit rate to 75.3% and reduces mishits by 67.1%. When U-Net is trained on more and higher-quality annotations, even better results can be expected.

**Patient summary:**

Kidney stones can be treated by applying shockwaves to the outside of the body. Ultrasound scans of the kidney are used to guide the machine delivering the shockwaves, but the shockwaves can still miss the stone. We used artificial intelligence to improve the accuracy in hitting the stone being treated.

## Take Home Message

This pilot study shows the feasibility of using artificial intelligence to improve extracorporeal shockwave lithotripsy (ESWL) treatment of kidney stones. The results suggest that an algorithm trained and tested with a neural network can improve the ESWL efficacy.

## 1 Introduction

Urolithiasis is an increasingly common condition that imposes a substantial burden on both patients and health care providers [1], [2]. The prevalence of urolithiasis varies globally, ranging from 4% to 20% [3], [4], [5]. Since Chaussy et al [6] reported extracorporeal shockwave lithotripsy (ESWL) treatment for urolithiasis in 1980, it has become the treatment option most utilized. The ability of shockwaves to fragment stones is the basis for ESWL and the efficacy depends on shockwaves hitting the stones [7]. ESWL, percutaneous nephrolithotomy (PCNL), and ureterorenoscopy/retrograde intrarenal surgery (URS/RIRS) are the main treatment options for symptomatic urolithiasis [8]. Of these, ESWL is the least invasive method with the fewest complications [9]. A global study covering a period of 20 yr found that the share of total treatments increased by 17% for URS/RIRS, remained the same for PCNL, and decreased by 14.5% for ESWL [10]. Another study investigating literature trends for urolithiasis treatments revealed that papers on URS/RIRS and PCNL increased by 171% and 279%, respectively, while papers on ESWL decreased by 17% [11].

Increases in ESWL efficacy should reduce retreatment rates, operating room time, anesthesia needs, endoscopic equipment use, and complication rates, thereby significantly reducing health care costs.

Since the creation of the computer there has been a desire to design computers capable of competing with human intelligence. This is achieved by imitating human cognitive function, a concept referred to as artificial intelligence (AI). Machine learning (ML) is a type of AI that learns through experience [12]. Several non-ML algorithms for tracking urinary stones have been developed and tested, but none has been widely adopted in clinical practice. It has been demonstrated that ML algorithms have the ability to outperform clinicians in image analysis [13], [14], [15].

**Fig. 1:**
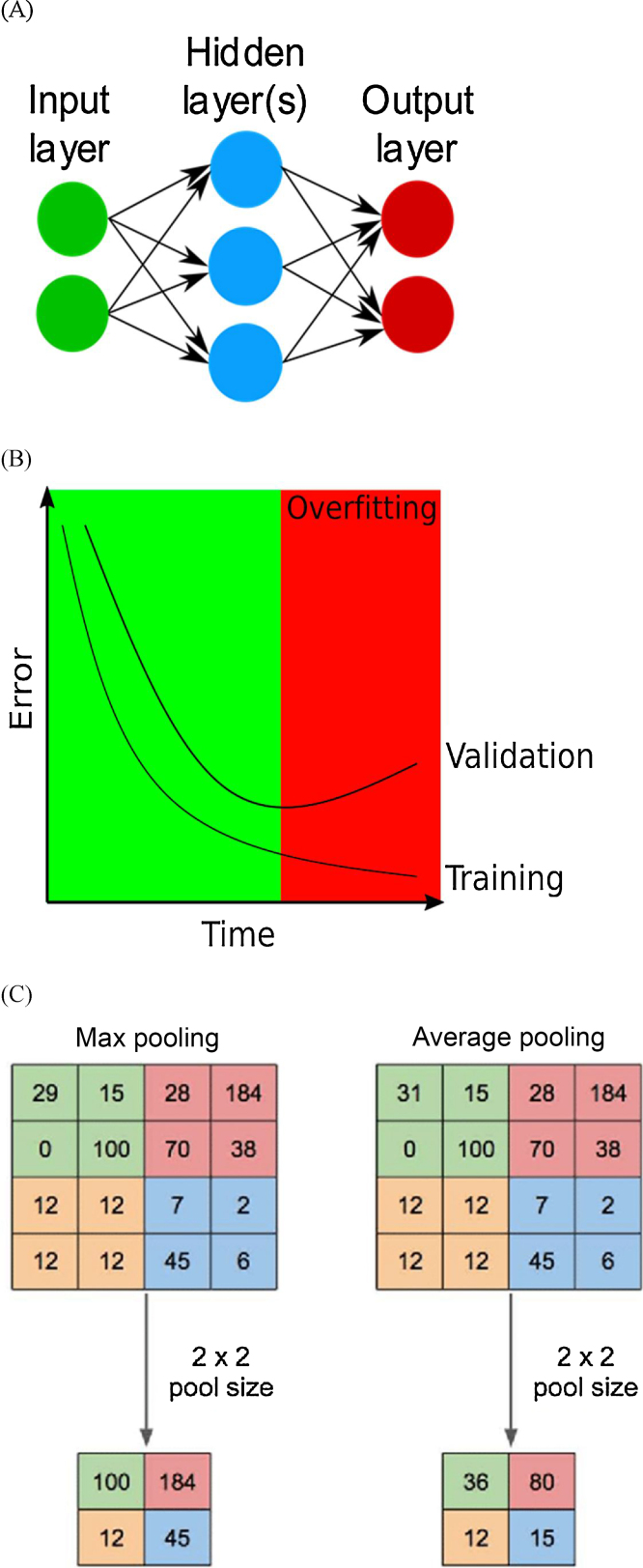
(A) Example of the architecture of simple neural network comprising an input layer with two nodes, a hidden layer with three nodes, and an output layer with two nodes. Created with Inkscape. (B) A graph describing overfitting. The training error continually decreases during training, eventually reaching zero if the model is trained for long enough. When overfitting starts, the validation error will start to increase because the model is getting worse at generalizing. The optimal stopping time is the lowest point on the validation curve. Based on a graph by Tretyakov [25]. (C) Image from Yani et al [26] (Creative Commons Attribution 3.0 license) showing that maximum pooling and average pooling downsample the input. In maximum pooling, the input is divided into parts and the highest value for each part gives the output. In average pooling, the average value for each part gives the output.

In supervised learning an algorithm is given labeled data such as ultrasound images of kidneys with stones and without stones to train it to differentiate “stone” images from “no stone” images [16], [17]. Popular ML algorithms inspired by biological neural circuits include artificial neural networks (NNs) (Fig. 1A). The first layer in an NN is called the input layer, and its role is to distribute the original input data to the next layer [18]. The output layer modifies input into the final output for the whole network, deciding whether an image contains a urinary stone or not in our example. Between the input and output layers there are “hidden” layers that are composed of weights that can be taught to handle complex problems [18]. How the connections and layers are structured defines the architecture of the NN [12], [16].

NN training is typically achieved using an optimizer that seeks to minimize a loss function through backpropagation. The role of the loss function is to measure the ability of the algorithm to model the given data (eg, to identify renal stones) and its value is used to update the network weights in order to minimize the error. To investigate the generalizability of the NN, it should be validated using different data from the data used for training. The validation loss is monitored during training: as the network improves, the validation error decreases with the training error. However, a common problem during training is overfitting (Fig. 1B), which is typically a result of the model memorizing the training data [19]. The result is a model that does not learn generalizable features, often identified by divergence of the validation loss. To prevent this, different training strategies are employed, such as early stopping and regularization. More importantly, a third independent data set, often referred to as the test set, is needed and used after the training procedure. The test set is used to measure the ability of the network to solve its task for unseen independent data.

**Fig. 2:**
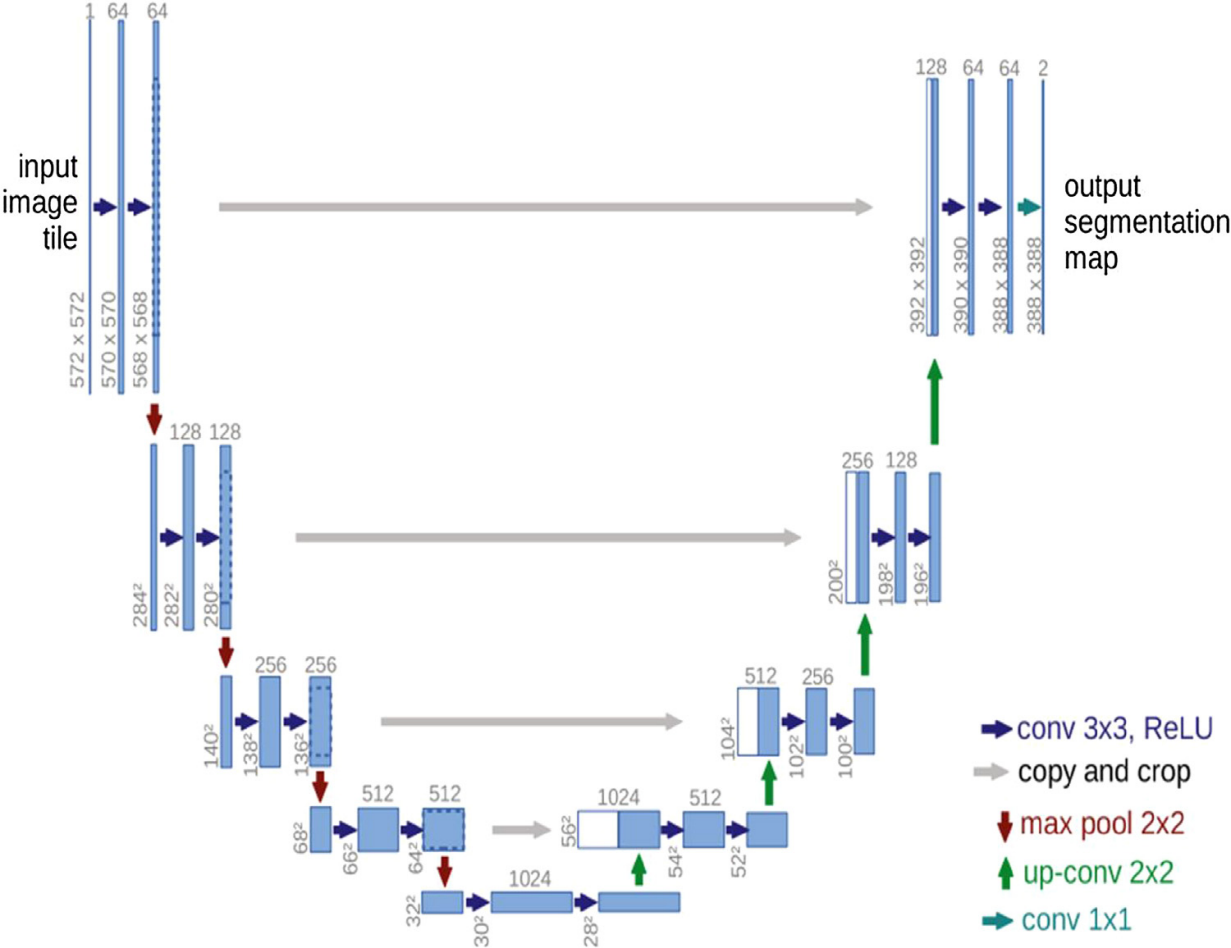
The original U-Net architecture created by Ronneberger et al [27]. Blue rectangles represent feature maps, while white rectangles represent feature maps copied via crossover connections. The arrows denote operators (dark and light blue = convolution; grey = crossover connection; red = maximum pooling; green = upsampling).

A convolutional NN (CNN) is preferred for complex image analysis [20]. CNNs are built to first identify features of low complexity, and then find features of higher complexity in deeper layers [20]. Convolution operations identify the essential features of the input (eg, lines or circles) and give outputs called feature maps. Pooling operations then downsample (reduce the resolution) of the feature maps to reduce the need for computational power in subsequent operations. Two of the pooling operations most often used are maximum pooling and average pooling, as explained in Figure 1C. When an algorithm performs segmentation of an image, it partitions it into semantic objects [20], such as determining which part of an image depicts a urinary stone [16]. Different CNNs have been constructed for segmentation purposes, one example of which is U-Net [20]. The first U-Net stage is downsampling, in which convolutional layers identify image features, while maximum pooling operators downsample the feature maps. In the last stage, which is upsampling, the feature maps are upsampled by upsampling operators and combined with copies of symmetric feature maps from the downsampling stage [20]. With these crossover connections, high-resolution features are preserved, as demonstrated in Figure 2.

## 2 Patients and methods

**Fig. 3:**
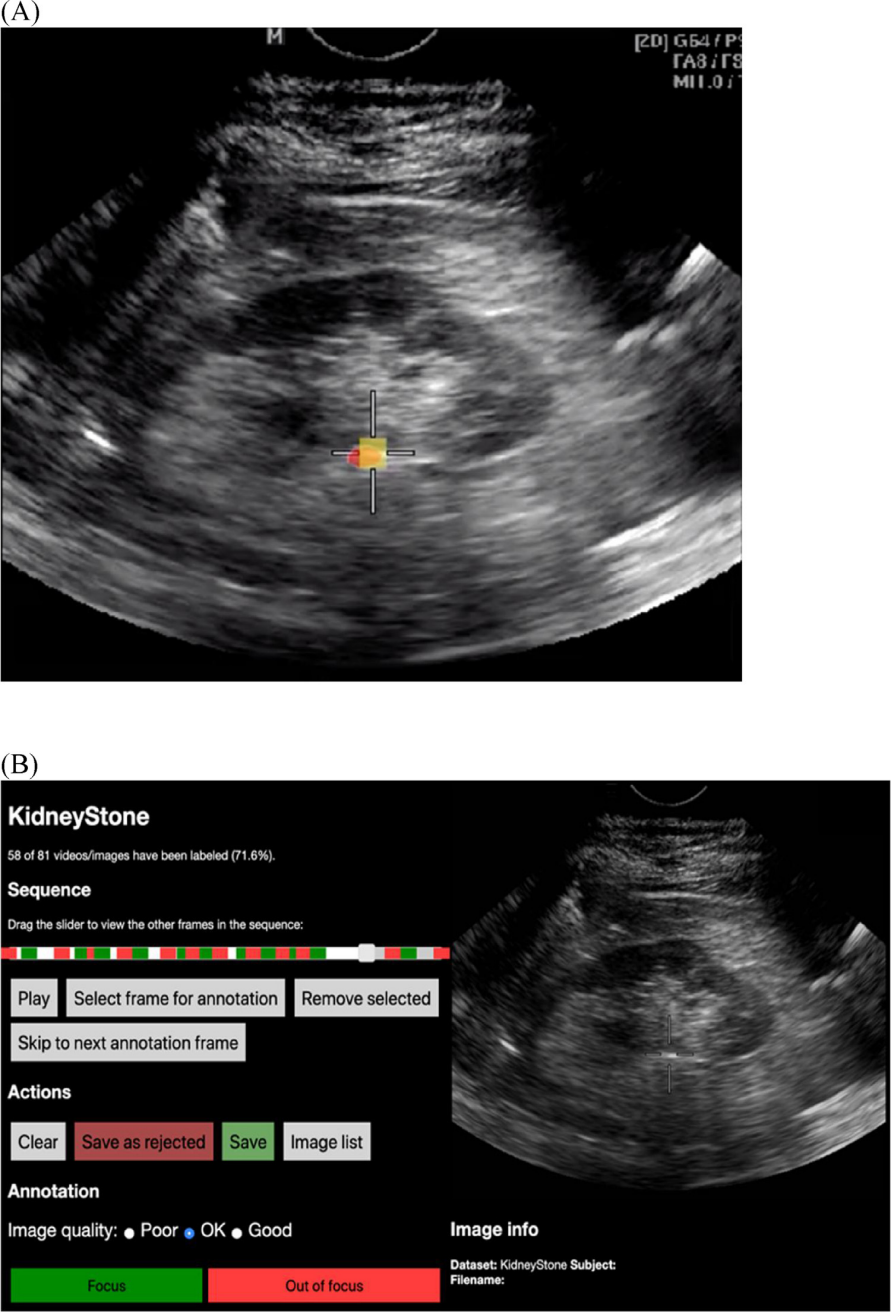
(A) Example of a frame for which the model reported that the stone was in focus, as ≥50% of the predicted stone (red) was within the focal zone (yellow). (B) Screenshot of the annotation tool. The ultrasound video with crosshairs is shown to the right, and a slider is used to go through the frames. To annotate a frame, the annotator clicks “Select frame for annotation” and chooses to label the frame as either “focus” or “out of focus”. If the stone is in focus for this frame, the annotator then continues the video and stops to label the first frame for which the stone goes out of focus. The frames between these two labels are automatically labeled “focus”. The green and red rectangles represent the frames labeled by the annotator.

Two-dimensional ultrasound images were analyzed to estimate the hit rate for operator-controlled ESWL and test the U-Net performance. To obtain images, a frame-grabber was attached to the ESWL machine (PiezoLith 3000, Richard Wolf GmbH, Knittlingen, Germany) for capture of inline real-time ultrasound images during ESWL. Each video was 30 min long and 5-min video sequences were randomly chosen for annotation. The annotator extracted ultrasound samples to label each frame as either “focus” when the stone was in the focal zone (FZ) or “out of focus” when the stone was not in the FZ (Fig. 3A). This process was carried out using an annotation tool (Fig. 3B). As a stone is usually in the FZ or out of the FZ for more than two consecutive frames, the annotation process was simplified by labeling only the transition points for intervals of frames. For example, if the first frame is labeled as in focus and the transition to out of focus occurs in the tenth frame, then all frames from the start until the tenth frame are classified as in focus.

During annotation we found that some stones were not visible in the ultrasound images, and these patients (cases 1, 3, and 9) were not included in the analysis of the hit rate for operator-controlled ESWL. In total, 731 frames were annotated directly, leading to a total of 23 212 frames. As the ultrasound device captures 15 frames/s, we ended up with 26 min of annotated ultrasound video, representing an average of 3.2 min for each patient. In addition, a second annotator delineated the kidney and renal stones in arbitrary frames for all patients. This resulted in binary masks for kidneys and kidney stones from a total of 57 images.

To test a standard U-Net convolutional network in kidney stone segmentation, it was trained using the delineated images. For training and validation of the network, we provided annotations of both the kidney and the kidney stone. Marking the kidney gave the algorithm a reference point or contextual information for where the stone should be, as a kidney stone remains approximately in the same position inside the kidney throughout the treatment. We conducted patient-based cross-validation. A total of 11 models were created by training on frames from nine patients and validating on frames from one patient. Of these 11 models, eight were tested on the same 23 212 frames annotated as in focus” or out of focus in eight patients.

The first outcome we wanted to investigate was the hit rate for operator-controlled ESWL continuously firing 90 pulses/min. The hit rate refers to the percentage of shockwaves that hit the stone, in this study defined as a shot for which more than 50% of the stone is in the FZ. To calculate this we needed to know the number of frames in which the stone was in focus out of a certain number of frames. Each frame was manually assigned a label of 0 (out of focus) or 1 (focus) by one observer and the sum of the labels gives the number of frames for which the stone is in the FZ. Using the R environment for statistical programming (www.r-project.org), the median hit rate for each patient with 95% confidence interval (CI) was estimated using bias-corrected and accelerated bootstrapping to evaluate the robustness of the results beyond sample estimates. The hit rate distribution among patients was examined by producing a histogram, boxplot, and a normal Q-Q plot in SPSS, and by performing a Shapiro-Wilk test and analysis of kurtosis and skewness. A χ^2^ test for goodness of fit was performed manually to determine whether the hit rates were uniformly distributed and to ultimately decide whether pooling was appropriate or not. A *p* value <0.05 was regarded as statistically significant and would lead to rejection of the null hypothesis that the hit rate distribution is uniform. The median hit rate with 95% CI was calculated on a frame level if pooling was appropriate, or on a patient level if pooling was inappropriate. The overall median hit rate with 95% CI was estimated via bias-corrected and accelerated bootstrapping.

To estimate the performance of the U-Net algorithm, the data were input to R to create a confusion matrix (Table 1) for which the ground truth was the annotated data. Frames for which the algorithm did not detect a stone were not included in the confusion matrix. R was then used to calculate classical metrics for the performance of classification models: accuracy, sensitivity, specificity, positive predictive value (PPV), negative predictive value (NPV), prevalence, detection rate, detection prevalence, balanced accuracy, Youden’s *J* statistic, the no-information rate, and Cohen’s κ. An explanation of the values is provided in Table 2. We then estimated the treatment time for U-Net-controlled ESWL relative to operator-controlled ESWL by dividing the number of frames annotated as in focus by the number of true positives. By multiplying the relative treatment time by the number of true negatives and dividing this by the number of frames annotated as out of focus, we estimated how U-Net would affect the number of mishits. Hits per minute was calculated for both operator-controlled ESWL and U-Net-controlled ESWL given a shockwave rate of 90/min. The median hit rate and 95% CI were calculated for each patient via bias-corrected and accelerated bootstrapping of 5000 samples of frames in R (Table 3).

**Table 1:** Confusion matrix design and test data (images annotated as in focus or out of focus) organized in a confusion matrix

	In focus (annotator)	Out of focus (annotator)	Total
**Design**			
In focus (AI)	TP	FP	TP + FP
Out of focus (AI)	FN	TN	FN + TN
Total	TP + FN	FP + TN	TP + FP + FN + TN
**Test data**			
In focus (AI)	5987	1961	7948
Out of focus (AI)	4700	5792	10 492
Total	10 687	7753	18 440

AI = artificial intelligence; TP = true positive; FP = false positive; FN = false negative; TN = true negative.

**Table 2:** Overview of the most important statistics describing performance of a classifier

Statistic	Definition
Accuracy	$\frac{\text{T}\text{P}+\text{T}\text{N}}{\text{T}\text{P}+\text{F}\text{P}+\text{T}\text{N}+\text{F}\text{N}}$
Sensitivity	$\frac{\text{T}\text{P}}{\text{T}\text{P}+\text{F}\text{N}}$
Specificity	$\frac{\text{T}\text{N}}{\text{T}\text{N}+\text{F}\text{P}}$
Positive predictive value (PPV)	$\frac{\text{T}\text{P}}{\text{T}\text{P}+\text{F}\text{P}}$
Negative predictive value (NPV)	$\frac{\text{T}\text{N}}{\text{T}\text{N}+\text{F}\text{N}}$
Prevalence	$\frac{\text{T}\text{P}+\text{F}\text{N}}{\text{T}\text{P}+\text{F}\text{P}+\text{F}\text{N}+\text{T}\text{N}}$
Detection rate	$\frac{\text{T}\text{P}}{\text{T}\text{P}+\text{F}\text{P}+\text{F}\text{N}+\text{T}\text{N}}$
Detection prevalence	$\frac{\text{T}\text{P}+\text{F}\text{P}}{\text{T}\text{P}+\text{F}\text{P}+\text{F}\text{N}+\text{T}\text{N}}$
Balanced accuracy	$\frac{\text{S}\text{e}\text{n}\text{s}\text{i}\text{t}\text{i}\text{v}\text{i}\text{t}\text{y}+\text{S}\text{p}\text{e}\text{c}\text{i}\text{f}\text{i}\text{c}\text{i}\text{t}\text{y}}{2}$
Youden’s *J* statistic	Sensitivity+Specificity-1
No-information rate:	
If $\left(TP+FN\right)>(\text{F}\text{P}+\text{T}\text{N})$	$\frac{\text{T}\text{P}+\text{F}\text{N}}{\text{T}\text{P}+\text{F}\text{P}+\text{F}\text{N}+\text{T}\text{N}}$
If $\left(FP+TN\right)>(\text{T}\text{P}+\text{F}\text{N})$	$\frac{\text{F}\text{P}+\text{T}\text{N}}{\text{T}\text{P}+\text{F}\text{P}+\text{F}\text{N}+\text{T}\text{N}}$

**Table 3:** Median hit rates for operator-controlled extracorporeal shockwave lithotripsy for each patient as estimated via bootstrapping

Patient	Frames in focus (*n*)	Total frames (*N*)	Median hit rate, % (95% CI)
1	–	–	–
2	1588	2974	53.4 (51.6–55.1)
3	–	–	–
4	1414	2397	59.0 (57.0–61.0)
5	1774	2798	63.4 (61.6–65.2)
6	1851	3382	54.7 (53.0–56.4)
7	1697	3544	47.9 (46.2–49.5)
8	2082	3926	53.1 (51.5–54.6)
9	–	–	–
10	789	3699	21.3 (20.0–22.7)
11	438	492	89.0 (86.2–91.7)
Total	11 633	23 212	55.2 (43.2–67.3)

CI = confidence interval.

Written permission to use anonymized ultrasound videos downloaded from patient records was obtained after evaluation by the regional ethics committee (reference number 2014/2261).

## 3 Results

**Fig. 4:**
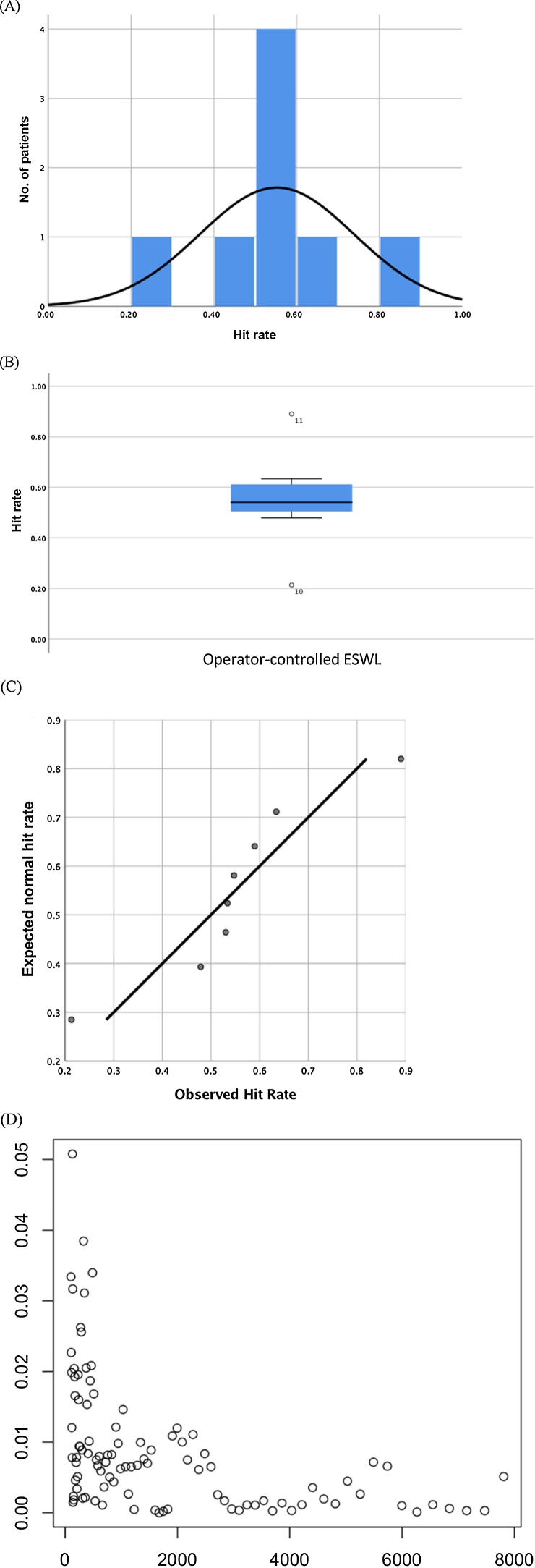
(A) Histogram of the hit rate for operator-controlled extracorporeal shockwave lithotripsy (ESWL). The distribution resembles a normal distribution, albeit with a degree of kurtosis. However, the kurtosis *z* value was not statistically significant. (B) Boxplot of the hit rate (*y*-axis) for operator-controlled ESWL (*x*-axis), showing an approximately symmetrical distribution consistent with a normal distribution. Patients 10 and 11 are outliers. (C) A normal Q-Q plot of the hit rate for operator-controlled ESWL. The points are close to the line, which typically indicates a normal distribution. Nevertheless, there seems to be a trend for how the points are organized around the line, suggesting the distribution might not actually be normal. (D) Convergence of the 95th percentile for the hit rate. The relative difference between two consecutive values tends towards zero as the number of bootstrapping iterations increases. It is only possible to extract 6435 different samples from the original sample size of eight. This limits how many samples we can bootstrap, as increasing the number of bootstrap samples increases the likelihood of extracting the same sample multiple times. To find the optimal number of bootstrap samples, we explored how many bootstrap samples it would take to stabilize the 95th percentile. This is shown in the graph, with number of bootstrap samples on the x-axis and the change of the 95th percentile in percentage on the y-axis. It is evident that the change is <1% after bootstrapping of 2000–3000 samples, indicating that the optimal number of bootstrap samples is 2000–3000.

The hit rate among patients was normally distributed, as shown in Figure 4A–D. This was supported by analysis of skewness (*z* = −0.005) and kurtosis (*z* = 1.73), and a Shapiro-Wilk test (*p* >  0.05). A χ^2^ goodness-of-fit test was then performed manually and controlled afterwards in R. The expected hit rate for each patient was calculated by multiplying the total number of frames for that patient by the pooled mean hit rate (50.12%). A χ^2^ value of 927.4 with seven degrees of freedom gave a *p* value <0.05, meaning the null hypothesis of a uniform distribution among the patients was rejected and the data should therefore not be pooled. By bootstrapping the median hit rates for eight patients with 3000 samples in R, we found a median hit rate of 55.2% (standard deviation 18.6%, 95% CI 43.2–67.3%). We chose to bootstrap with 3000 samples on the basis of a convergence analysis of the 95th percentile, as explained in Figure 4D.

The algorithm was unable to find a stone in 20.6% of the frames, so they were not included in the analysis. For the remaining 18 440 frames, the degree of overlap between the predicted stone area and the FZ was calculated. Overlap of ≥50% was considered “in focus”. The test results were organized in a confusion matrix in R using the annotator as the ground truth and the performance was calculated (Table 1). The algorithm found that 58.0% (prevalence) of the frames had stones in focus (Table 4). The accuracy of the algorithm was 63.9%, meaning that it correctly classified 63.9% of the frames as either “in focus” or “out of focus”. Of the frames with stones in focus, the algorithm was able to classify approximately half as “in focus”, as the sensitivity was 56.0%. The algorithm was better at classifying the stones that were “out of focus”, with specificity of 74.7%. The PPV (the number of frames that the algorithm correctly classified as “in focus”) was 75.3% and the NPV (the number of frames the algorithm correctly classified as “out of focus”) was55.2%. Note that the PPV corresponds to the hit rate if the lithotripter fires shockwaves in accordance with the algorithm. The detection rate was 32.5%, while the detection prevalence was considerably higher at 43.1%, indicative of a substantial number of false positives (when AI classifies a frame as “in focus” when the stone is actually “out of focus”). With a Youden’s *J* statistic of 30.7% (criterion: >0), Cohen’s κ of 0.2931 (criterion: >0), and a no-information rate of 58.0% (lower than accuracy), the algorithm performance is better than randomly guessing whether stones are in or out of focus, suggesting that it can correctly track kidney stones in ultrasound images. The treatment time relative to operator-controlled ESWL was 1.94 (11 633/5 987), while the mishit rate was 32.9% ([1.94 × 1961]/[23 212 – 11 633]) of the rate for operator-controlled ESWL. Operator-controlled ESWL hits the stone 45 times per minute (90/min × 11 633/23 212), while U-Net-controlled ESWL hit the stone 23 times per minute (90/min × 5987/23 212).

**Table 4:** Calculated performance statistics for a U-Net model when tested on ultrasound images annotated as in or out of focus

Statistic	Value
Accuracy (%)	63.9
Sensitivity (%)	56.0
Specificity (%)	74.7
Positive predictive value (%)	75.3
Negative predictive value (%)	55.2
Prevalence (%)	58.0
Detection rate (%)	32.5
Detection prevalence (%)	43.1
Balanced accuracy (%)	65.4
Youden’s *J* statistic (%)	30.7
No-information rate (%)	58.0
Cohen’s κ	0.2931

## 4 Discussion

Our findings suggest that there is significant potential to optimize the ESWL hit rate, as we estimated an operator-controlled hit rate of 55.2% could be improved to 75.3% using a U-Net neural network to control ESWL and the total number of shockwaves missing the stone would be reduced to approximately one-third, ultimately making the procedure safer for patients.

There are several limitations and weaknesses to the way in which we estimated the hit rate. First, the annotator (a medical student) was inexperienced in ultrasound image interpretation; and second, identifying the exact borders of the stone was difficult because of low image resolution, which we experienced as a significant issue during annotation. The resolution was low because of the quality of the probe-scanner system itself and because the probe had to be retracted during shockwave firing. A future solution could be to register pre-intervention computed tomography (CT) images with the ultrasound images, which would probably make it easier for the annotator to make correct annotations by suggesting the stone position relative to the kidney.

Another problem is that the ultrasound images we sampled were from the first 5 min of treatment. During treatment the stone is progressively fragmented and therefore becomes more difficult to identify (also true for fluoroscopy) and to subsequently hit, so the samples we used are not representative of the whole treatment course. However, when a stone becomes too difficult to identify it is not relevant for our analysis, as the annotator cannot decide whether the stone is in focus or not. Estimated hit rates between patients were normally distributed, suggesting that they are representative. Our definition of a shockwave hit as 50% overlap between the stone and the FZ may not be optimal, as marginal hits may also contribute to fragmentation, resulting in underestimation of the hit rate. A bias might have been introduced when we excluded patients 1, 3, and 9 because of the lack of visibility of their stones on ultrasound. For operator-controlled ESWL, the operator would also not be able to localize their stones on ultrasound, so periodic fluoroscopy would be needed. Consequently, the operator has less control of the real-time location of a stone and it would probably spend more time out of focus. When images from these patients are left out, the operator-controlled hit rate might be overestimated.

The training and performance testing of the algorithm also have several limitations and weaknesses. The algorithm was trained and validated on data without crosshairs annotated by a second inexperienced observer. Thus, the training set might contain false-positive stones, limiting the potential of the algorithm to learn stone-tracking correctly. Some of the training and validation annotations were performed on ultrasound images in which the stone was difficult to identify (including patients 1, 3, and 9), increasing the probability of false-positive stones.

The algorithm was only trained on 57 images from a total of 11 patients. The training set was clearly not large enough for optimizing the algorithm efficacy, and the algorithm has significant potential for improvement if more patients are included and an experienced radiologist uses CT to provide accurate annotations. As in the estimation of the operator-controlled hit rate, estimation of overlap is also an issue in the performance test. The test set was annotated by a medical student who assessed whether the stone was in focus or not via a semi-subjective visual evaluation of the stone and FZ overlap. By contrast, the algorithm was trained on images in which the stones were delineated. When the stone edges are marked by hand, computer software can calculate the stone and FZ overlap much more accurately than a human visually evaluating the overlap. As a result, although the test set annotator and the algorithm might be in perfect agreement over the location of a stone in a test set image, they might estimate different degrees of stone-FZ overlap, resulting in disagreement on whether a stone is in focus or not. This especially relates to stones that are close to 50% within the FZ. In these cases, even small differences in estimation of the overlap might influence the decision on “in focus” versus “out of focus”. This results in more uncertainty in the metrics describing the algorithm performance.

Using two different inexperienced annotators has some additional weaknesses. The algorithm first learns what one of the annotators interprets as stones and is then tested on what the other annotator interprets as stones. One problem here is interobserver variability, which we confirmed was significant: comparison of the two annotators revealed a mismatch rate of 37.5%. This means that the algorithm will never perform perfectly on the test set, as the annotators for the training and test sets disagreed over the definition of stone borders. In fact, accounting for interobserver variability instead of only using one observer strengthens the confidence in our metrics indicating that the algorithm has stone-tracking ability.

Should the algorithm become better than the test set annotator at identifying stones, the metrics would underestimate the performance of the algorithm. To see if the algorithm performed significantly better than the metrics implied, we visually examined several of the ultrasound videos of algorithm-predicted stones and tested the trained algorithm on the same type of annotations used in the training set. After reviewing the results, the idea that the algorithm clearly outperformed the test set annotations was rejected.

We discussed treating frames in which the algorithm did not detect a stone as though the algorithm reported that the stone was “out of focus”. This would lead to improvements in all the AI performance parameters except for a decrease in sensitivity (51.2%). Most notably, we saw increases in accuracy to 67.0%, specificity to 83.0%, and Youden’s *J* statistic to 34.2%. The argument for analyzing the data in this way is that stones that are not detected will not be shot at, resulting in a lower risk of treatment complications. Having said that, we chose not to do this, as we could not control for whether frames in which stones were not detected by the algorithm had stones or not, which would have resulted in over-rating of the tracking ability of the algorithm. In addition, it would not affect the PPV, which is arguably the most important parameter when analyzing the algorithm performance at the current state of the project.

We were able to identify three studies that estimated ESWL hit rates between 40% and 60% [13], [21], [22]. Our estimated hit rate of 55.2% is at the higher end of range compared to the other studies, but our wide 95% CI (43.2–67.3%) fits well with their observations. Different definitions of the hit rate and small sample sizes limit the generalizability of these studies.

To date there are no publications on ML algorithms used to localize urinary stones in ultrasound images for ESWL treatment. Singla et al [23] tried to localize urinary stones using fluoroscopy during ESWL treatment with RetinaNet and achieved precision of 70% ± 10% using a different ML algorithm.

**Fig. 5:**
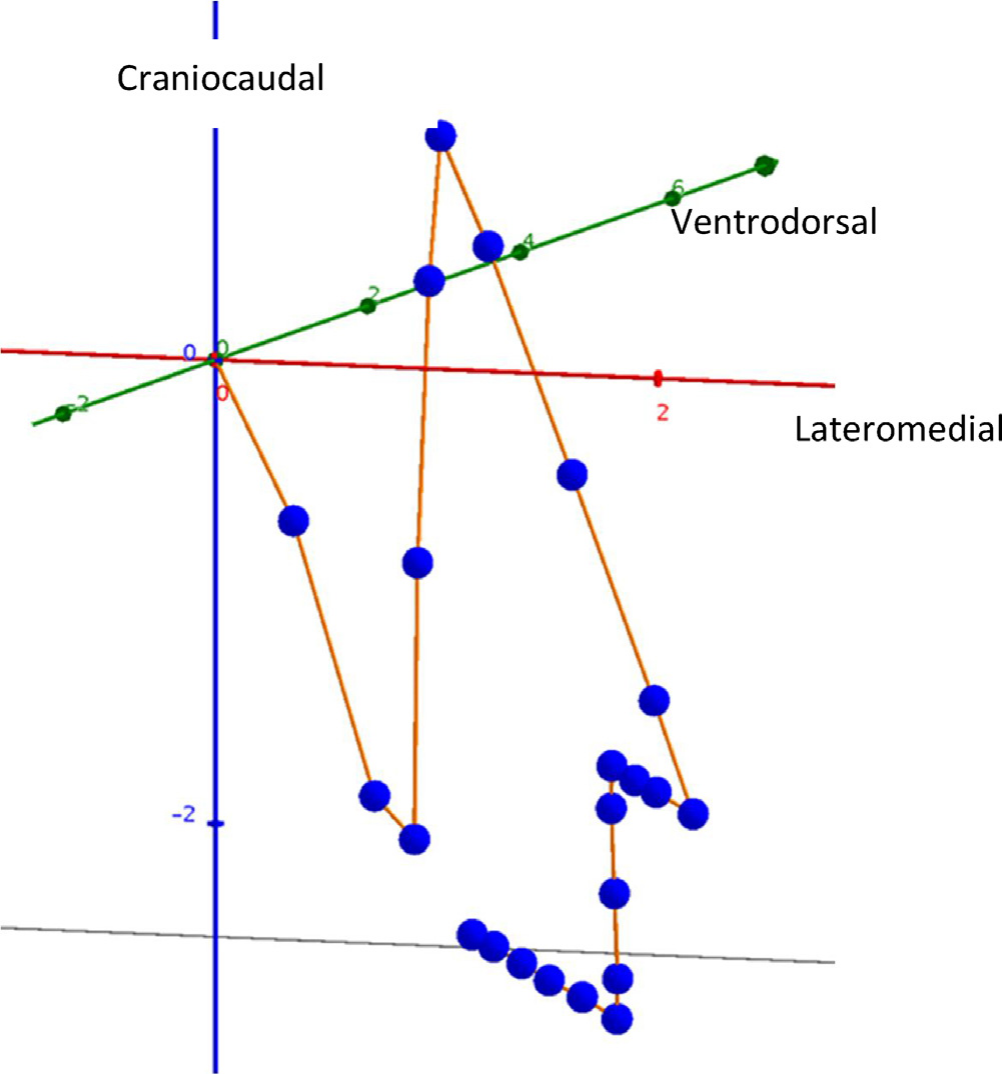
A graph by Kragset [28] demonstrating the three-dimensional movement of a urinary stone during one respiratory cycle. Each dot represents the stone’s location at a specific time point. When lines between the dots are long, the movement is large. The dots at the end of expiration are very close to each other, meaning the stone is almost standing still – this is the optimal time interval to target the stone.

Our algorithm can be implemented by stopping the lithotripter from firing shockwaves when the stone is out of focus. An algorithm similar to that used by Singla et al [23] could also be added to create a pipeline that uses both ultrasound and fluoroscopy, which could potentially further improve stone-tracking ability. It has been shown that treatment pulse rates of 60–90 yield the best stone-free rate, but it should be noted that this rate is based on testing of different constant rates, regardless of whether the stone is within the focal zone or not [24]. Current ESWL treatment routines use approximately 3000–4000 pulses per treatment at a hit rate of 50%, resulting in approximately 2000 hits. Algorithm-controlled ESWL may only require 2000 shockwaves, which would thus lead to a reduction in treatment time. In fact, shockwave rates could be increased so that when the stone passes through the focal zone it could be hit multiple times. Previous unpublished findings by our group showed that the stone is relatively stationary at the end of expiration (Fig. 5) [25]. This physiological fact could be much better utilized in algorithm-controlled ESWL, with shockwaves fired at a higher rate while the stone is stationary inside the focal zone at the end of each expiration. The algorithm accounts for the entire kidney image, and not just the stone itself, so another potential benefit of algorithm-controlled ESWL is that the hit rate can be maintained in the later stages of the treatment course when the stone often becomes unclear on both ultrasound and fluoroscopy. Before the algorithm is implemented in clinical practice it should be trained and tested on more and higher-quality annotations, preferably by a uroradiologist using information from pretreatment CT. Annotation of training sets should also be carried out at several different institutions to improve the generalizability of the ML algorithm.

## 5 Conclusions

We estimated an operator-controlled ESWL hit rate of 55.2% (95% CI 43.2–67.3%), which means that approximately half of the shockwaves applied miss the stone. Algorithm-controlled ESWL increased the hit rate to approximately 75.3% and reduced the total number of shockwaves missing the stone by approximately 67.1%. Our results indicate that a U-Net neural network trained and tested on better annotations will be able to improve ESWL efficacy.

  ***Author contributions:*** Carl-Jørgen Arum had full access to all the data in the study and takes responsibility for the integrity of the data and the accuracy of the data analysis.

  *Study concept and design:* Muller, Østvik, Langø, Arum.

*Acquisition of data:* Muller, Abildsnes, Østvik, Kragset, Gangås, Birke.

*Analysis and interpretation of data:* Muller, Abildsnes, Østvik, Kragset, Langø, Arum.

*Drafting of the manuscript:* Muller, Abildsnes, Østvik, Kragset, Langø, Arum.

*Critical revision of the manuscript for important intellectual content:* All authors.

*Statistical analysis:* Muller, Østvik.

*Obtaining funding:* Langø, Arum.

*Administrative, technical, or material support:* None.

*Supervision:* Langø, Arum.

*Other:* None.

  ***Financial disclosures:*** Carl-Jørgen Arum certifies that all conflicts of interest, including specific financial interests and relationships and affiliations relevant to the subject matter or materials discussed in the manuscript (eg, employment/affiliation, grants or funding, consultancies, honoraria, stock ownership or options, expert testimony, royalties, or patents filed, received, or pending), are the following: None.

  ***Funding/Support and role of the sponsor:*** This project was supported by the 10.13039/501100004590Central Norway Regional Health Authority, Stiftelsen SINTEF, the Faculty of Medicine and Health Sciences, and the Norwegian National Advisory Unit for Ultrasound and Image-Guided Therapy at St. Olavs Hospital in Trondheim, Norway. The sponsors played no direct role in the study.
